# Epigenetic reprogramming of human lung cancer cells with the extract of bovine parthenogenetic oocytes

**DOI:** 10.1111/jcmm.12306

**Published:** 2014-05-30

**Authors:** Zhenfei Wang, Rinuo Dao, Luri Bao, Yanhua Dong, Haiyang Wang, Pengyong Han, Yongli Yue, Haiquan Yu

**Affiliations:** The Key Laboratory of Mammal Reproductive Biology and Biotechnology, Ministry of Education, Inner Mongolia UniversityHuhhot, China

**Keywords:** lung cancer, reprogramming, tumour suppressor gene, oncogenic gene, extract, parthenogenetic oocyte

## Abstract

The tumour suppressor gene silencing and proto-oncogene activation caused by epigenetic alterations plays an important role in the initiation and progression of cancer. Re-establishing the balance between the expression of tumour suppressor genes and proto-oncogenes by epigenetic modulation is a promising strategy for cancer treatment. In this study, we investigated whether cancer cells can be epigenetically reprogrammed by oocyte extract. H460 human lung cancer cells were reversibly permeabilized and incubated with the extract of bovine parthenogenetic oocytes. Bisulphite sequencing showed that bovine parthenogenetic oocyte extract induced significant demethylation at the promoters of the tumour suppressor genes *RUNX3* and *CDH1*, but not at the promoter of the oncogenic pluripotency gene *SOX2*. Chromatin immunoprecipitation showed that the histone modifications at *RUNX3* and *CDH1* promoters were modulated towards a transcriptionally activating state, while those at *SOX2* promoter towards a transcriptionally repressive state. Correspondingly, bovine parthenogenetic oocyte extract reversed the epigenetic silencing of *RUNX3* and *CDH1*, and repressed the expression of *SOX2*. At the functional level, proliferation, anchorage-independent growth, migration and invasion of H460 cells was strongly inhibited. These results indicate that bovine parthenogenetic oocyte extract changes the expression patterns of tumour suppressor and oncogenic genes in cancer cells by remodelling the epigenetic modifications at their promoters. Bovine parthenogenetic oocyte extract may provide a useful tool for epigenetically reprogramming cancer cells and for dissecting the epigenetic mechanisms involved in tumorigenesis.

## Introduction

The precise regulation of gene expression is vital for maintenance of cell homoeostasis. Coordinated expression of tumour suppressor genes and proto-oncogenes controls the orderly process of cell proliferation, differentiation and death. Under-expression of tumour suppressor genes and overexpression of proto-oncogenes leads to the initiation and progression of cancer. Epigenetic alterations, especially those in DNA methylation and histone modifications, play an important role in disrupting the balance of tumour suppressor gene and proto-oncogene expression [1–3]. In cancer cells, many tumour suppressor genes are abnormally silenced by DNA hypermethylation, increase of repressive histone modifications, and decrease of activating histone modifications at their promoters; while many proto-oncogenes are abnormally activated by DNA hypomethylation, decrease of repressive histone modifications and increase of activating histone modifications at their promoters [1–3]. Unlike genetic mutations, epigenetic alterations are reversible. Therefore, remodelling the epigenetic modifications at the promoters of tumour suppressor genes and proto-oncogenes in cancer cells is a promising way to restore the balance of their expression, and thus reprogramme cancer cells towards normal cells [3].

DNA methyltransferase inhibitors and histone deacetylase inhibitors are widely used epigenetic modifiers, which can activate epigenetically silenced tumour suppressor genes by reducing DNA methylation and increasing histone acetylation at their promoters. However, these drugs work by general inhibition of the corresponding epigenetic enzymes, so they generate demethylating and acetylating effects on whole chromatin rather than on the chromatin regions with abnormal structures [1–3]. This action mechanism leads to a lack of specificity. Besides activating the transcription of tumour suppressor genes, these drugs also up-regulate the expression of oncogenic genes [4–7]. For example, treatment of human acute lymphoblastic leukaemia cells with DNA methyltransferase inhibitor 5-aza-2-deoxycytidine leads to hypomethylation of LINE-1 repeat elements and thus triggers its retrotransposition, which subsequently activates the expression of proto-oncogene c-MET [4]. Treatment of rat chondrosarcoma cells with 5-aza-2-deoxycytidine induces promoter hypomethylation of the oncogenic pluripotency gene *SOX2* and up-regulates its transcription [5]. Furthermore, treatment of human breast cancer cells with histone deacetylase inhibitor VPA activates Wnt signalling by up-regulating β-catenin expression, which promotes the expansion of cancer stem cells [7]. The non-specific effects of these drugs pose a risk for their clinical application [5–7]. Therefore, it is necessary to develop new more safe approaches for epigenetic reprogramming of cancer cells.

Studies on the development of fertilized oocytes and nuclear transfer embryos demonstrate that ooplasm has potent reprogramming capacity. The large number of reprogramming factors stored in ooplasm can efficiently remodel the epigenetic modifications of sperm and donor cell chromatin [8,9]. The activity of these reprogramming factors is retained in oocyte extract and can be used to remodel the epigenetic modifications of normal somatic cells. For example, treatment of reversibly permeabilized fibroblasts with bovine MII and parthenogenetic oocyte extract significantly decreased the H3K18Ac level of the somatic chromatin [10]. Similarly, incubating reversibly permeabilized ovine [11] and swine [12] fibroblasts in Xenopus oocyte extract induced significant reduction in H3K9me and DNA methylation levels of the somatic nuclei. The oocyte extract-induced epigenetic remodelling is mediated by soluble reprogramming factors, which travel into permeabilized cells during incubation and directly change the epigenetic modifications in specific chromatin regions [13–17]. This system provides a useful pathway for reprogramming cell fate. However, existing studies have focused on reprogramming normal somatic cells, whereas none of them has investigated whether the reprogramming capability of oocyte extracts can be used to reverse the epigenetic alterations and re-establish normal gene expression patterns in cancer cells.

By Using H460 human lung cancer cells and bovine parthenogenetic oocyte extract, we studied whether cancer cells can be reprogrammed by incubation with oocyte extract. Bovine oocyte extract treatment remodelled the epigenetic modifications at both tumour suppressor gene and oncogenic pluripotency gene promoters, leading to activation of the epigenetically silenced tumour suppressor genes and repression of the oncogenic gene *SOX2*. Furthermore, the treatment effectively inhibited the malignant cancer cell phenotype, suggesting potential therapeutic applications for bovine parthenogenetic oocyte extracts.

## Material and methods

### Bovine oocyte collection, maturation and parthenogenetic activation

Bovine oocyte collection, *in vitro* maturation and parthenogenetic activation were performed as previously described [18].

### Bovine oocyte extract preparation

Bovine oocyte extract was prepared as described previously with minor changes [16]. Briefly, parthenogenetically activated oocytes were digested with 0.5% pronase for 5 min. to remove the zona pellucida. The zona-free oocytes were transferred into 1.5 ml tubes and washed three times with extraction buffer (5 mM MgCl_2_, 60 mM NaCl, 2 mM β-mercaptoethanol, protease inhibitor cocktail, 5 mM EGTA and 50 mM HEPES, pH 7.4) containing an energy regenerating system (2 mM ATP, 20 mM phosphocreatine, 20 U/ml creatine kinase and 2 mM GTP). The washed oocytes were resuspended (100 oocytes/μl) in extraction buffer containing the energy regenerating system and disrupted by centrifugation twice at 20,800 × *g* for 30 min. at 4°C. Under this condition, the oocytes were completely disrupted as demonstrated by phase-contrast microscopy (Figure S1). The lysate was mixed by pipetting and centrifuged at 5000 × *g* for 10 min. at 4°C, and the supernatant was harvested as the oocyte extract.

### Oocyte extract treatment

The human non-small cell lung cancer cell line NCI-H460 was purchased from Cell Bank, China Academy of Sciences (Shanghai, China), and cultured in RPMI-1640 medium supplemented with 10% FBS at 37°C at 5% CO_2_. To permeabilize the cells, 2 × 10^6^ H460 cells were suspended in 20 μg/ml digitonin solution for 2 min. The permeabilized cells were suspended in bovine oocyte extract (5000 cells/10 μl extract; extract-treated cells) or an equal volume of extraction buffer (buffer-treated cells) at 38.5°C for 3.5 hrs with occasional tapping. For membrane resealing, the cell suspension was diluted with 1 ml RPMI-1640 containing 2 mM CaCl_2_ and incubated for 2 hrs at 37°C. After pelleting by centrifugation at 400 × *g* for 5 min., the cells were subjected to normal culture or used for assays.

### Bisulphite sequencing

DNA was isolated from H460 cells by using the Wizard SV Genomic DNA Purification System (Promega, Southampton, UK). Bisulphite conversion was performed with the EZ DNA Methylation kit (Zymo Research, Orange, CA, USA) according to the manufacturer's recommendations. Bisulphite converted DNA was amplified with the primers listed in Table S1. The PCR products were then cloned into the vector pEASY-T1 (Transgene, Beijing, China), and at least 10 clones from each sample were subjected to DNA sequencing.

### Chromatin immunoprecipitation (ChIP) assay

Chromatin immunoprecipitation assays were performed with the ChIP-IT™ Chromatin Immunoprecipitation kit (Active Motif, Carlsbad, CA, USA) according to the manufacturer's protocol. The antibodies used were as follows: mouse anti-trimethyl Lys27 of histone H3 (anti-H3K27me3, Abcam, Ab6002), rabbit anti-trimethyl Lys9 of histone H3 (anti-H3K9me3, Abcam, Ab8898), rabbit anti-acetyl Lys9 of histone H3 (anti-H3K9ac, Upstate, 07-352) and rabbit anti-trimethyl Lys4 of histone H3 (anti-H3K4me3, Upstate, 07-473). DNA recovered from the immunoprecipitates and pre-IP chromatin (input) was quantified by real-time PCR with the following primers: *RUNX3* promoter, forward 5′-ATC CGGGCTCTGGGCACTCG-3′ and reverse 5′-AAGCGGTTGCAGTGGGCGTG-3′; *CDH1* promoter, forward 5′-CCCCTCTCAGTGGCGTCGGA-3′ and reverse 5′-CGAGAGGCTGCGGCTCCAAG-3′; and *SOX2* promoter, forward 5′-GAGAAGGGCGTGAGAGAGTG-3′ and reverse 5′-AAACAGCCAGTGC AGG AGTT-3′. The PCR condition was denaturation at 95°C for 30 sec., and amplification by 40 cycles of 95°C for 5 sec. and 60°C for 31 sec., followed by melting curve analysis. The results were reported as the ratio of immunoprecipitated DNA to input DNA.

### Quantitative real-time PCR

Total RNA was isolated from cells using RNAiso Reagent (TaKaRa, Dalian, China) and reverse transcribed by using the PrimeScript RT reagent kit (TaKaRa). Real-time PCR was performed with the primers shown in Table S2 and SYBR Premix Ex *Taq* (TaKaRa) under the same condition as that described above on a 7300 Real-Time PCR System (Applied Biosystems, Foster City, CA, USA). The mean relative expression levels of each gene were calculated from triplicate samples by the ΔΔCt method by using *GAPDH* as an endogenous control.

### Western blot analysis

Cells were disrupted in lysis buffer (AR0105-30; Boster, Wuhan, China) supplemented with 0.5 mM phenylmethylsulfonyl fluoride, 10 μg/ml leupeptin, 5 μg/ml pepstatin and 2.1 μg/ml aprotinin. Protein lysates were separated by 10% sodium dodecyl sulphate-polyacrylamide gel electrophoresis and transferred to polyvinylidene difluoride membranes. The membranes were incubated with rabbit antibody to RUNX3 (1:1000; ab49117; Abcam, Cambridge, UK), rabbit antibody to E-cadherin (1:1000; ab53033; Abcam), SOX2 (1:1000; 20118-1-AP; Proteintech, Chicago, IL, USA) or mouse antibody to β-Actin (1:2000; 60008; Proteintech), and subsequently incubated with HRP-conjugated goat anti-rabbit IgG (BA1054; Boster) or HRP-conjugated goat antimouse IgG (BA1050; Boster). Signals were detected using an enhanced chemiluminescence kit (Pierce, Rockford, IL, USA).

### 3-(4,5-dimethylthiazol-2-yl)-2,5-diphenyltetrazolium bromide (MTT) assay

Cell proliferation was measured by MTT assay. Cells were seeded in 96-well plates in 200 μl complete RPMI-1640 medium and cultured at 37°C. At different time-points, MTT solution (5 mg/ml) was added and incubated at 37°C for 4 hrs. Then, the medium was absorbed off and 100 μl dimethyl sulfoxide was added to each well. Following vibrating on a shaker for 10 min., the plates were measured for absorbance at 490 nm wavelength by using a microtiter plate reader (Bio-Rad 550, Hercules, CA, USA).

### Soft agar assay

A 0.6% (wt/vol) solution of low melting point agar was prepared in normal medium, placed into six-well culture plates, and allowed to solidify. A layer of 0.3% low melting point agar containing 1 × 10^4^ cells was gently pipetted on top. The cells were incubated in a humidified atmosphere (5% CO_2_) at 37°C, and the number of colonies larger than 100 μm was counted after 3 weeks.

### Cell migration and invasion assay

Migration and invasion assays were performed with 24-well Transwell chambers (polycarbonate membrane, 8 μm pore size; Costar, Cambridge, MA, USA). For the migration assay, 4 × 10^4^ cells suspended in 200 μl serum-free RPMI-1640 were added into each upper chamber, and 600 μl of RPMI-1640 containing 20% FCS was added into each lower chamber as a chemoattractant. The plates were incubated for 24 hrs at 37°C, and then the media was removed from the Transwell chambers and the cells on the upper surface of the Transwell membrane were wiped off. Cells that had migrated to the lower surface of the Transwell membrane were fixed and stained with haematoxylin, and the number of cells in five randomly selected fields at ×200 magnification were counted.

For the invasion assay, the upper surfaces of the Transwell membranes were pre-coated with Matrigel (diluted 1:7; BD Biosciences, San Jose, CA, USA), which was allowed to solidify at 37°C for 4 hrs. Then the cells were added to the Transwell membranes and the assay was conducted using the same method as the migration assay.

### Statistical analysis

Bisulphite sequencing data were analysed by using the chi-squared test; ChIP and real-time PCR data were analysed by using one-way anova; data from the MTT assay, soft agar assay, cell migration assay and cell invasion assay were analysed by using the unpaired Student's *t*-test. Significance was accepted when *P* < 0.05.

## Results

### Bovine oocyte extract demethylates *RUNX3* and *CDH1* promoters but not *SOX2* promoter

**Fig. 1:**
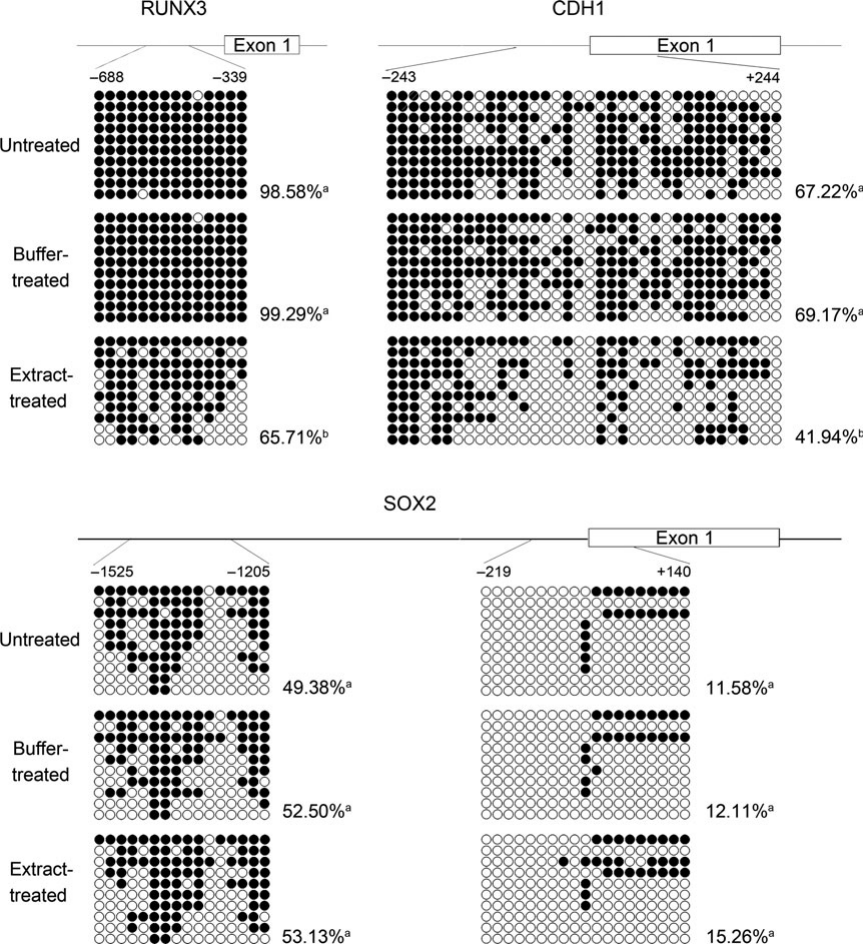
Treatment of H460 cells with bovine oocyte extract leads to demethylation of the *RUNX3* and *CDH1* promoters but not of the *SOX2* promoter. Schematics indicate the CpG islands of *RUNX3* and *CDH1* promoters and the regions targeted for bisulphite sequencing at *SOX2* promoter. The open circles (○) represent unmethylated CpG sites and closed circles (•) represent methylated CpG sites. The methylation level of each region is presented as the number of methylated CpG sites as a percentage of the total number of unmethylated and methylated CpG sites. a *versus* a: *P* > 0.05; a *versus* b: *P* < 0.05.

To determine whether bovine oocyte extract can affect the DNA methylation levels of gene promoters in cancer cells, we performed bisulphite sequencing for the tumour suppressor genes *RUNX3* and *CDH1*, and the oncogenic pluripotency gene *SOX2*. The results (Fig. 1) showed that the promoter CpG islands of *RUNX3* and *CDH1* were both hypermethylated in untreated and buffer-treated cells. For extract-treated cells, bovine oocyte extract induced significant demethylation of the CpG islands. The methylation levels decreased by 33.81% and 39.36% for *RUNX3* and *CDH1* respectively*,* compared to those of buffer-treated cells. Differently, the methylation level of *SOX2* promoter was similar in untreated, buffer-treated and extract-treated cells. These results suggest that bovine oocyte extract can specifically reverse the CpG island hypermethylation of tumour suppressor gene promoters in cancer cells.

### Bovine oocyte extract remodels the histone modification patterns at *RUNX3*, *CDH1* and *SOX2* promoters

**Fig. 2:**
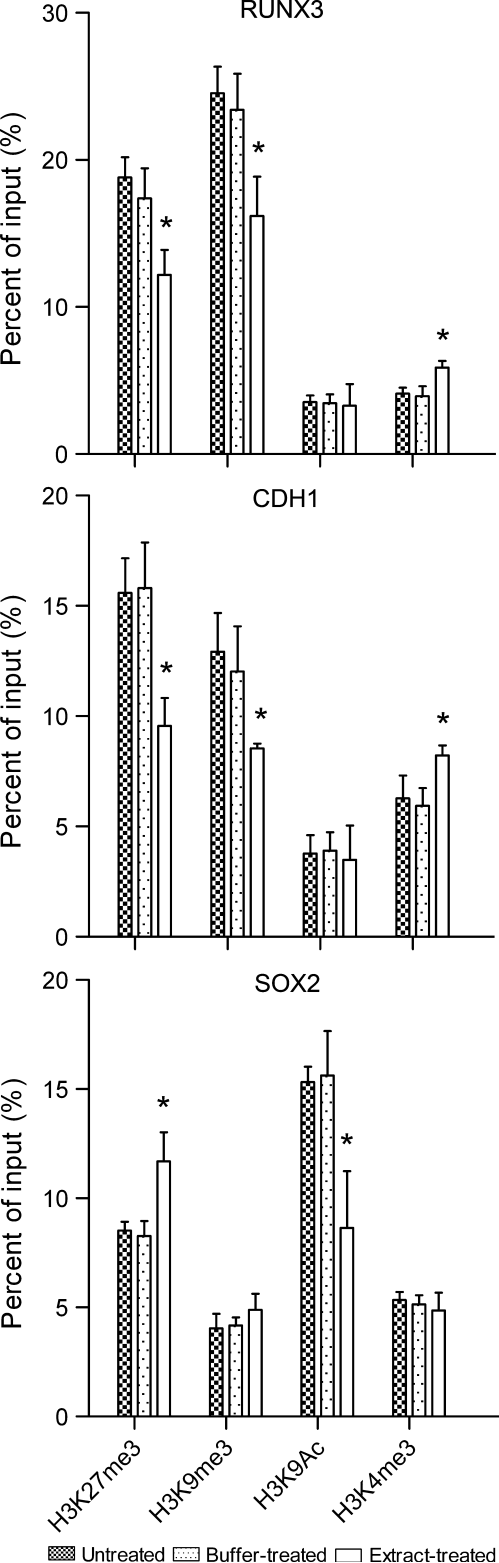
Treatment with bovine oocyte extract alters the histone modification patterns at the promoters of *RUNX3*, *CDH1* and *SOX2* in H460 cells. Results are shown for ChIP analysis of the indicated repressive (H3K27me3 and H3K9me3) and activating (H3K9Ac and H3K4me3) histone modifications. Error bars represent the standard deviation (SD, *n* = 3); *P < 0.05 *versus* untreated and buffer-treated cells.

To determine whether extract treatment on H460 cells affects histone modifications, ChIP assays were performed following extract treatment. For both the *RUNX3* and *CDH1* promoters, the repressive histone modifications H3K27me3 and H3K9me3 were significantly reduced, and the activating histone modification H3K4me3 was significantly increased in extract-treated cells as compared to untreated and buffer-treated cells (Fig. 2). Conversely, for the *SOX2* promoters, the repressive histone modification H3K27me3 was significantly increased and the activating histone modification H3K9Ac was significantly reduced in extract-treated cells as compared to untreated and buffer-treated cells (Fig. 2). These results suggest that treatment of cancer cells with bovine oocyte extract can generate different remodelling effects on the histone modifications of tumour suppressor gene and oncogenic gene promoters.

### Bovine oocyte extract activates the expression of *RUNX3* and *CDH1*, and decreases the expression of *SOX2*

**Fig. 3:**
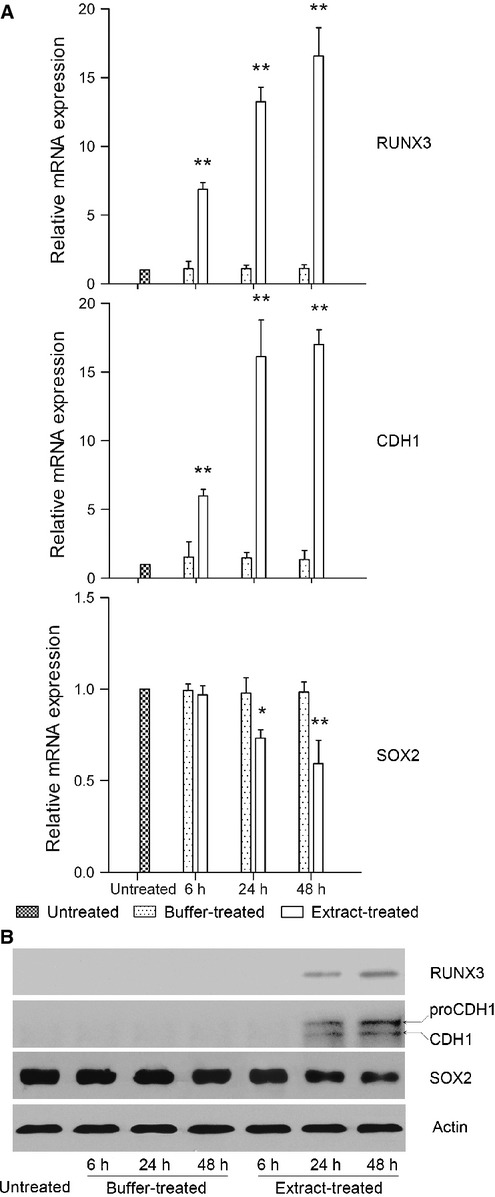
Treatment with bovine oocyte extract activates the expression of *RUNX3* and *CDH1*, and represses the expression of *SOX2* at both mRNA (**A**) and protein (**B**) levels. Cells were treated, returned to culture for 6, 24 or 48 hrs, and then subjected to real-time PCR (**A**) and Western blot (**B**) analyses. Error bars represent the SD (*n* = 3); *P < 0.05 *versus* untreated and buffer-treated cells, **P < 0.01 *versus* untreated and buffer-treated cells. Two forms of CDH1 were detected by Western blot. Based on published reports [33,34], the lower molecular weight band (∼105 kD) is mature CDH1, and the higher molecular weight band (∼120 kD) represents the precursor, proCDH1.

We next examined whether the epigenetic remodelling of DNA methylation and histone modifications by bovine oocyte extract changes the expression of *RUNX3, CDH1* and *SOX2*. As shown in Figure 3, the expression of *RUNX3* and *CDH1* was silenced in both untreated and buffer-treated cells. However, in extract-treated cells, *RUNX3* and *CDH1* mRNA expression was significantly increased at 6 hrs, and further increased at 24 and 48 hrs after return to culture (Fig. 3A). The protein expression of RUNX3 and CDH1 began to appear in extract-treated cells at 24 hrs and was further enhanced at 48 hrs after return to culture (Fig. 3B). In contrast, the mRNA and protein expression of *SOX2* was active in untreated and buffer-treated cells, but decreased in extract-treated cells after return to culture for 24 and 48 hrs (to 63.17 ± 11.67% and 49.79 ± 6.29%, respectively, of the buffer-treated cells by densitometry of western blots normalized to β-actin; Fig. 3). These results indicate that the bovine oocyte extract treatment effectively activates *RUNX3* and *CDH1* expression, and decreases *SOX2* expression in H460 cells.

### Bovine oocyte extract does not demethylate repetitive sequences

**Fig. 4:**
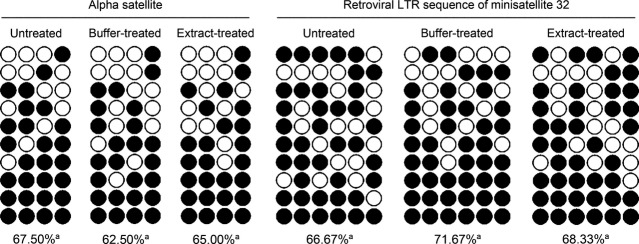
Treatment with bovine oocyte extract does not affect the DNA methylation levels of the alpha satellite and retroviral long terminal repeat (LTR) sequence of minisatellite MS32 in H460 cells. The open circles (○) represent unmethylated CpG sites, and closed circles (•) represent methylated CpG sites. The methylation level of each region is presented as the percentage of methylated CpG sites relative to the total number of unmethylated and methylated CpG sites. a *versus* a: *P* > 0.05.

To further confirm the specificity of bovine oocyte extract-induced demethylation, we examined the methylation status in H460 cells for repetitive sequences. Bisulphite sequencing demonstrated that the methylation levels of two repetitive sequences, alpha satellite sequence and retroviral long terminal repeat sequence of minisatellite MS32, were similar in untreated, buffer-treated and extract-treated cells (Fig. 4).

### Bovine oocyte extract inhibits the malignant phenotype of H460 cells

**Fig. 5:**
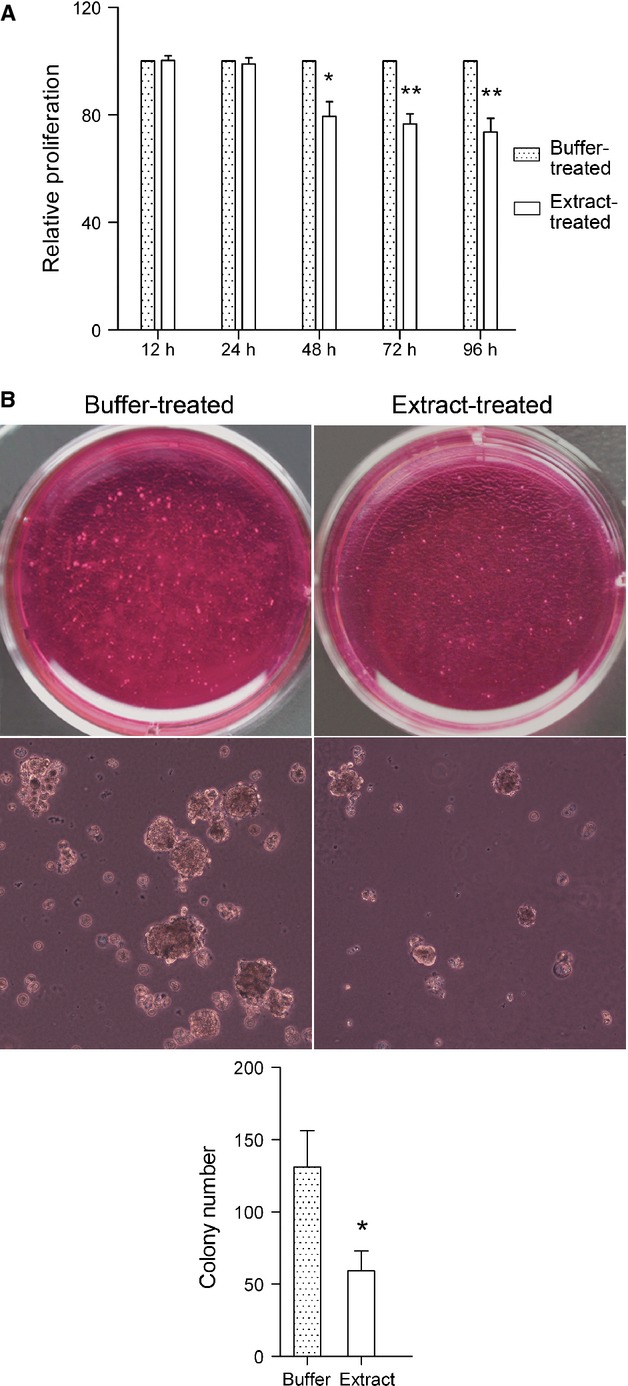
Treatment with bovine oocyte extract inhibits proliferation (**A**) and anchorage-independent growth (**B**) of H460 cells. Error bars represent the SD (*n* = 3); *P < 0.05 *versus* buffer-treated cells, **P < 0.01 *versus* buffer-treated cells; scale bar: 100 μm.

**Fig. 6:**
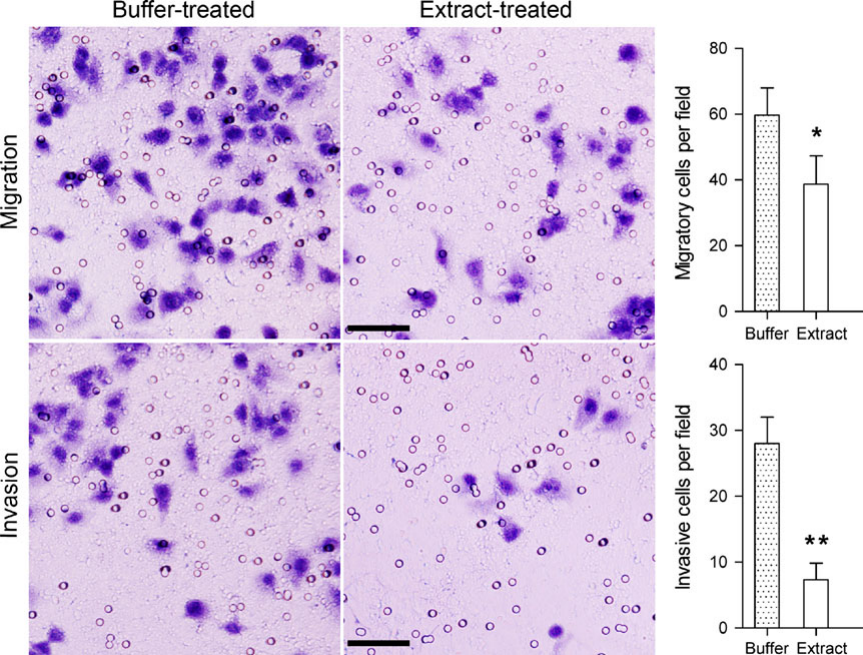
Treatment with bovine oocyte extract inhibits migration and Matrigel invasion by H460 cells. Error bars represent the SD (*n* = 3); *P < 0.05 *versus* buffer-treated cells, **P < 0.01 *versus* buffer-treated cells; scale bars: 50 μm.

We next examined whether oocyte extract affects the cancerous phenotype of H460 cells. In MTT assays, extract-treated cells exhibited slower proliferation than buffer-treated cells since 48 hrs after return to culture, and in soft agar assays, extract-treated cells formed significantly fewer and smaller colonies than buffer-treated cells (Fig. 5). Furthermore, in migration assays, the number of extract-treated cells that migrated through Transwell membranes was 35.20% lower than buffer-treated cells, and in invasion assays, the number of extract-treated cells that passed through the Matrigel barrier was 73.81% lower than buffer-treated cells (Fig. 6). These results indicate that bovine oocyte extract strongly inhibits the proliferation, anchorage-independent growth, migration and invasion of H460 cells.

## Discussion

The promoter CpG islands of tumour suppressor genes, which are hypomethylated in normal cells, are frequently hypermethylated in cancer cells. Such hypermethylation is an important mechanism for silencing tumour suppressor gene expression in human cancers. By using bisulphite sequencing, we showed that the promoter CpG islands of the tumour suppressor genes *RUNX3* and *CDH1* were hypermethylated in H460 cells, consistent with other reports [19,20]. Treatment of H460 cells with bovine oocyte extracts for 3.5 hrs significantly decreased the methylation levels of the CpG islands from both genes. Because the treatment time was short and the cells were suspended in a serum-free environment during incubation, the extract-induced demethylation is an active process that is independent of DNA replication. Many molecules expressed in the ooplasm are known to posses demethylation activity [8,21] and to be able to travel into permeabilized cells during extract incubation to directly catalyse demethylation reactions [13–15]. Therefore, the demethylation that we observed at the *RUNX3* and *CDH1* promoters might be catalysed by these demethylases.

Active demethylation reactions always occur at specific chromatin regions, rather than throughout whole chromatin [22]. In this study, bovine oocyte extract induced specific demethylation at the CpG islands of *RUNX3* and *CDH1* promoters, without decreasing the methylation level of *SOX2* promoter and two important repetitive sequences. Demethylation of the promoter of *SOX2* can up-regulate its expression, which promotes proliferation and invasion of a range of cancer cells [5,23–25]. Hypomethylation of repetitive sequences leads to genome instability, which enhances malignant phenotype of cancer cells [5]. The specificity for tumour suppressor gene promoters of bovine oocyte extract-induced demethylation suggests that it is a safe demethylation approach.

Cytosine nucleoside analogues, such as 5-azacytidine and 5-aza-2′-deoxycytidine, are currently the most widely used DNA methylation inhibitors. These drugs become incorporated into the DNA of proliferating cells during replication, where their modified cytosine rings form covalent complexes with DNA methyltransferases, leading to the depletion of active enzymes. Thus, DNA methylation levels are decreased after several rounds of DNA replication [1,3]. This passive demethylation by cytosine nucleoside analogues has two drawbacks: first, the incorporation of nucleoside analogues into DNA causes a permanent alteration of the genome, often leading to mutagenesis in surviving daughter cells [3,26]; second, nucleoside analogues can be incorporated into DNA only during the S-phase of mitotic process, so they have little effect on quiescent cells, such as cancer stem cells [1]. Notably, the demethylation by bovine oocyte extract is through the action of the active demethylation factors present in extract [13,15,17], thus avoiding the risk of causing mutations. Moreover, bovine oocyte extract induces demethylation independently of DNA replication, so it could potentially have effects on cancer stem cell populations.

Many molecules expressed in the ooplasm are able to modulate histone modifications. These include the Jumonji domain containing proteins (which can demethylate H3K27me3 and H3K9me3) [27,28], the mixed lineage leukaemia 2 (which can methylate H3K4) [29], the enhancer of zeste homologue 2 (which can methylate H3K27) [30], the histone deacetylase 1 and histone deacetylase 2 [28], and so on. Although it has been widely reported that oocyte extract treatment can change the overall levels of histone modifications of somatic cell chromatin [13,14,17], little is known about its effect on the histone modifications of specific genomic sequences. By using ChIP assay, we showed that the modulation of H3K27me3, H3K9me3, H3K9Ac and H3K4me3 by bovine oocyte extract was different at tumour suppressor gene and oncogenic gene promoters. Therefore, the histone modification modulators from oocyte extract do not interact randomly with the genome elements of H460 cells, but are selectively targeted to specific chromatin regions and remodel the local histone modification patterns.

DNA methylation and histone modifications play important roles in regulating chromatin structure. After bovine oocyte extract treatment, the DNA methylation and repressive histone modifications were reduced, and activating histone modification H3K4me3 was increased at the promoters of tumour suppressor genes *RUNX3* and *CDH1*. These epigenetic changes induce an open chromatin structure at *RUNX3* and *CDH1* promoters, facilitating their access by transcription factors and thus activating transcription. In contrast, at the promoter of oncogenic pluripotency gene *SOX2*, the repressive histone modification H3K27me3 was increased and the activating histone modification H3K9Ac was reduced. These epigenetic changes convert the chromatin structure at *SOX2* promoter towards a close state, hindering transcription factor recruitment and thereby inhibiting transcription. Therefore, bovine oocyte extract generates different epigenetic reprogramming effects on tumour suppressor genes and the oncogenic pluripotency gene *SOX2*.

*RUNX3* and *CDH1* are important tumour suppressor genes. RUNX3 has strong proliferation-suppressive function. It inhibits the oncogenic Wnt signalling pathway by forming a complex with the TCF4-β-catenin complex and preventing it from binding to target promoters such as the *c-Myc* and *cyclinD1* promoters [31]. CDH1 has potent migration and invasion suppressor roles by serving as a key transmembrane protein for the maintenance of intercellular junctions in epithelial tissues [28]. Moreover, CDH1 also functions as a negative regulator of cell proliferation by participating in Cdk inhibitor-dependent G1 arrest and DNA damage-induced G2 arrest [32]. *SOX2* is a pluripotency gene crucial to maintain the stemness of embryonic stem cells. Recent studies have shown that SOX2 also plays an important role in the progression of many malignancies [5,23–25]. Overexpression of SOX2 is found in adenocarcinoma, squamous cell, large-cell, and small-cell lung carcinomas, where it promotes tumorigenesis and maintenance of cancer stem cell characteristics [23,24]. Bovine oocyte extract effectively reversed the epigenetic silencing of *RUNX3* and *CDH1*, and repressed the expression of *SOX2* in H460 cells; correspondingly, proliferation, anchorage-independent growth, migration and invasion of H460 cells was inhibited. These results demonstrate that the epigenetic changes induced by bovine oocyte extract lead to functional changes in the cancerous phenotype of H460 cells, and suggest that these changes could be mediated by the activation of RUNX3 and CDH1, as well as the repression of SOX2.

In summary, bovine parthenogenetic oocyte extract treatment induces an activating epigenetic state at the promoters of the tumour suppressor genes *RUNX3* and *CDH1*, and a suppressing epigenetic state at the promoters of the oncogenic pluripotency gene *SOX2*. This epigenetic remodelling effect leads to activation of *RUNX3* and *CDH1* expression, and repression of *SOX2* expression. Consequently, the malignant phenotype of cancer cells can be strongly inhibited. Thus, bovine parthenogenetic oocyte extract treatment provides a new system for investigating the epigenetic mechanisms of cancer, and a promising approach for cancer epigenetic therapy.

## Supporting information

**Figure S1** Phase-contrast microscopy (100×) shows that the oocytes were completely disrupted after centrifugation.

**Table S1** Bisulphite sequencing primers.

**Table S2** Real-time PCR primers.
