# Supplementary material for: Epigenetic reprogramming of human lung cancer cells with the extract of bovine parthenogenetic oocytes
Source: J Cell Mol Med. 2014 May 30;18(9):1807–15. doi: 10.1111/jcmm.12306 (PMC4196656; doi:10.1111/jcmm.12306)
Supplement: Supplementary file 1 — Figure S1 Phase-contrast microscopy (100×) shows that the oocytes were completely disrupted after centrifugation. [file jcmm0018-1807-SD1.doc]

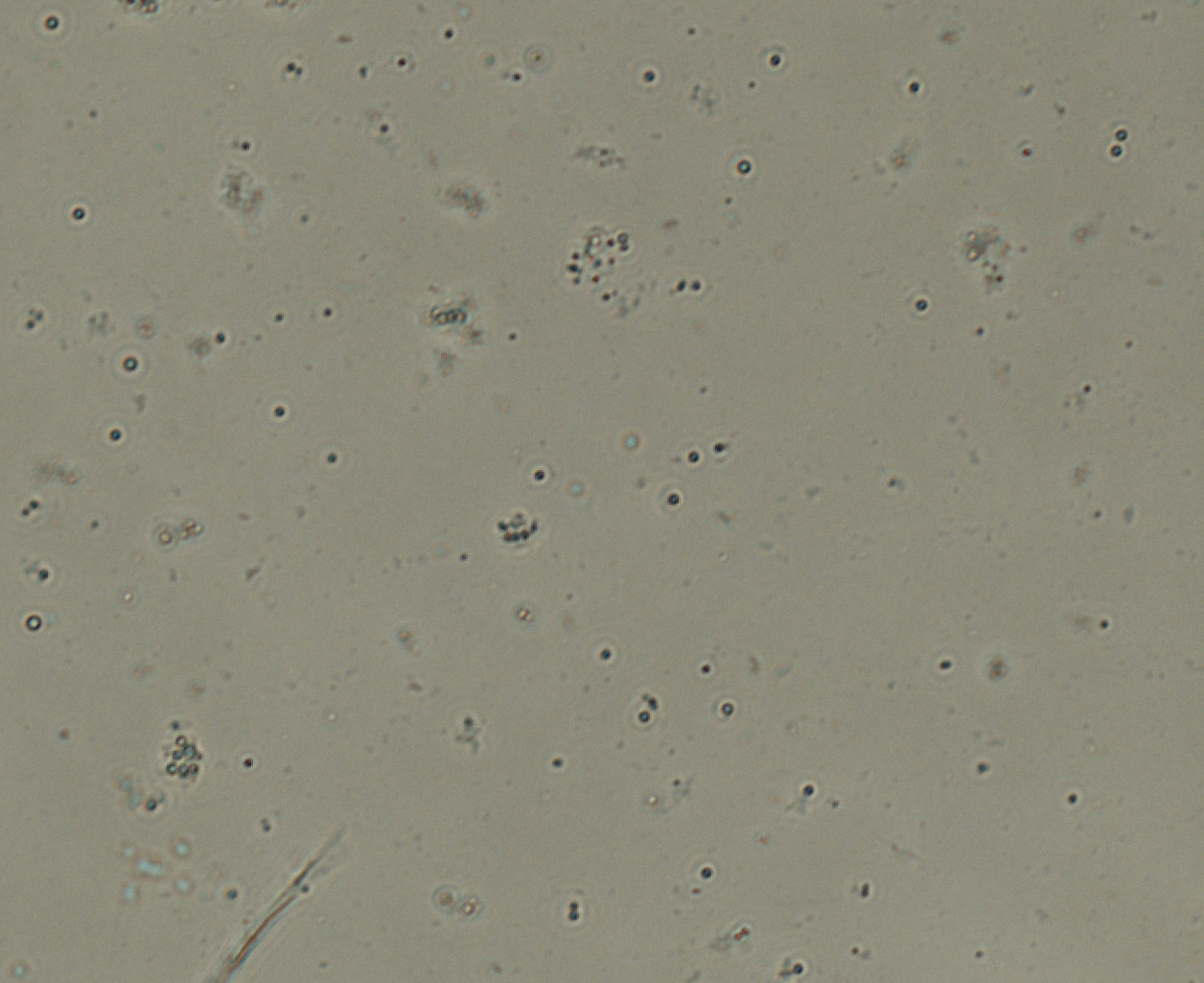


Supplementary Figure 1 Phase-contrast microscopy (100×) shows that the oocytes were completely disrupted after centrifugation.
