# Supplementary material for: Epigenetic reprogramming of human lung cancer cells with the extract of bovine parthenogenetic oocytes
Source: J Cell Mol Med. 2014 May 30;18(9):1807–15. doi: 10.1111/jcmm.12306 (PMC4196656; doi:10.1111/jcmm.12306)
Supplement: Supplementary file 2 — Table S1 Bisulphite sequencing primers. [file jcmm0018-1807-SD2.doc]

**Supplementary Table 1.** Bisulfite sequencing primers

| Gene  name | Forward primer (F)  Reverse primer (R) |
| --- | --- |
| *RUNX3* | F: TAGTTTTGTAGAGGGTTTTTTAGTG  R: AATTAAAACCAACATTAACCTAAAC |
| *CDH1* | F: AATAAAAGAATTTAGTTAAGTGT  R: GTTGTTGTTGTTGTAGGTATT |
| *SOX2*  *(-1525 ~ -1205)* | F: TTAATAAGAGAGTGGAAGGAAATTTAGA  R: CTCTCCAAACACAAATACAAACAAC |
| *SOX2*  *(-219 ~ +140)* | F: AAAGGTTTTTTAGTGGTTGGTAGGT  R: AAAACTCAAACTTCTCTCCCTTTCT |
| *Alpha satellites* | F: TAATTAATTAAACCCCTTT  R: TTTTTATGTTTAAGATTGG |
| *Retroviral LTR of*  *minisatellite MS32* | F: CCACTCAAACATAAATTTAA  R: GTTTAGATTGTGTAGTTTAA |
