# Supplementary material for: Epigenetic reprogramming of human lung cancer cells with the extract of bovine parthenogenetic oocytes
Source: J Cell Mol Med. 2014 May 30;18(9):1807–15. doi: 10.1111/jcmm.12306 (PMC4196656; doi:10.1111/jcmm.12306)
Supplement: Supplementary file 3 — Table S2 Real-time PCR primers. [file jcmm0018-1807-SD3.doc]

**Supplementary Table 2.** Real-time PCR primers

| Gene name | Forward primer (F)  Reverse primer (R) | GenBank  accession number |
| --- | --- | --- |
| *GAPDH* | F: TGTCCCCACTGCCAACGTGTCA  R: GCGTCAAAGGTGGAGGAGTGGGT | NM_002046 |
| *RUNX3* | F: CAGCACCACAAGCCACTTCA  R: GGTCGGAGAATGGGTTCAGTT | NM_004350 |
| *CDH1* | F: CGGGAATGCAGTTGAGGATC  R: AGGATGGTGTAAGCGATGGC | NM_004360 |
| *SOX2* | F: TACAGCATGTCCTACTCGCAG  R: GAGGAAGAGGTAACCACAGGG | NM_003106 |
